# Ruptured Intracranial Aneurysm Presenting as Isolated Acute Subdural Hemorrhage

**DOI:** 10.7759/cureus.28314

**Published:** 2022-08-23

**Authors:** Denis Babici, Phillip M Johansen, Stu L Newman, Evan Packer, Brian Snelling

**Affiliations:** 1 Neurology, Florida Atlantic University Charles E. Schmidt College of Medicine, Boca Raton, USA; 2 Neurosurgery, Boca Raton Regional Hospital, Boca Raton, USA

**Keywords:** open craniotomy, transarterial coil embolization, intracranial aneurism, mri images, spontaneous intracranial hemorrhage

## Abstract

Ruptured intracranial aneurysms are often associated with serious neurologic sequelae, often as a result of subarachnoid or intraparenchymal hemorrhage. Less commonly, ruptured intracranial aneurysms can lead to subdural hemorrhage. However, the characteristic clinical presentation and optimal treatment of associated subdural hemorrhage are unclear due to the paucity of such cases that exist in the current literature. Affected patients may complain of nonspecific symptoms such as headaches, nausea, and confusion. Because of the severity of the disease, rapid diagnosis and intervention is required to lower the high morbidity and mortality rates. Commonly used treatment options include endovascular coiling and microsurgical clipping. Neuroendovascular surgery is often preferred, especially in aneurysms not amenable to surgical clipping, in poor surgical candidates, and cases with endovascularly favorable anatomy. The authors present the case of a patient who came to the hospital with ischemic stroke-like symptoms and was found to have a ruptured posterior communicating artery (PCoA) aneurysm and associated acute subdural hematoma (SDH) without obvious subarachnoid hemorrhage (SAH). Endovascular coiling of the aneurysm was performed successfully the following craniotomy for SDH evacuation, and the patient was discharged to a rehabilitation facility

## Introduction

Ruptured intracranial aneurysms are often associated with subarachnoid, or less commonly intraparenchymal hemorrhage, depending on the aneurysm’s location. Additionally, the incidence of acute subdural hematoma (SDH) secondary to a ruptured intracranial aneurysm is only approximately 8%. However, the prevalence of an isolated subdural hematoma without intraparenchymal or subarachnoid hemorrhage (SAH) is significantly lower, making this an extremely rare finding [1]. Suspicion for an underlying vascular anomaly, particularly in the absence of a traumatic event or coagulopathy, should be high in the setting of an acute SDH. Rapid non-invasive analysis of the cerebrovasculature, such as magnetic resonance (MR) or computed tomography angiography (CTA), can frequently identify an underlying vascular abnormality and allow timely treatment to prevent rebleeding.

## Case presentation

**Figure 1:**
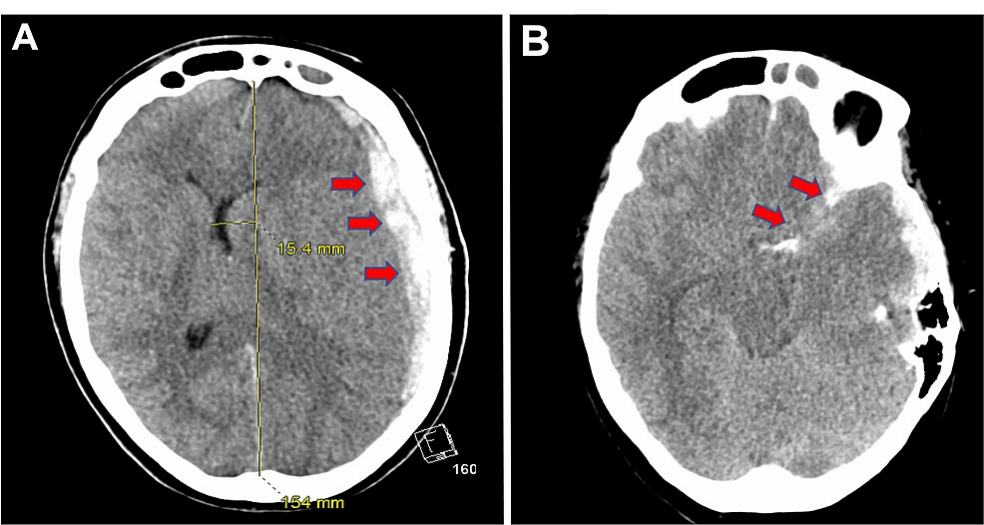
CT Scan of the Head A. Left hemispheric subdural hematoma resulting in mass effect, left-to-right midline shift, and brain herniation (red arrows). B. Scant subarachnoid hemorrhage in the Sylvian fissure (red arrows).

A 65-year-old female with no known past medical history suddenly developed right-sided weakness and leftward gaze deviation, prompting a stroke alert to be initiated in the field prior to ED arrival. Once in the ED, she became drowsy and was unable to follow commands. After several episodes of vomiting, she was emergently intubated for airway protection. Her initial blood pressure was 240/120 mmHg, for which intravenous labetalol and nicardipine were administered. A computed tomography (CT) of the head revealed a left-sided subdural hematoma with a left-to-right midline shift and scant SAH in the Sylvian fissure (Figure 1).

**Figure 2:**
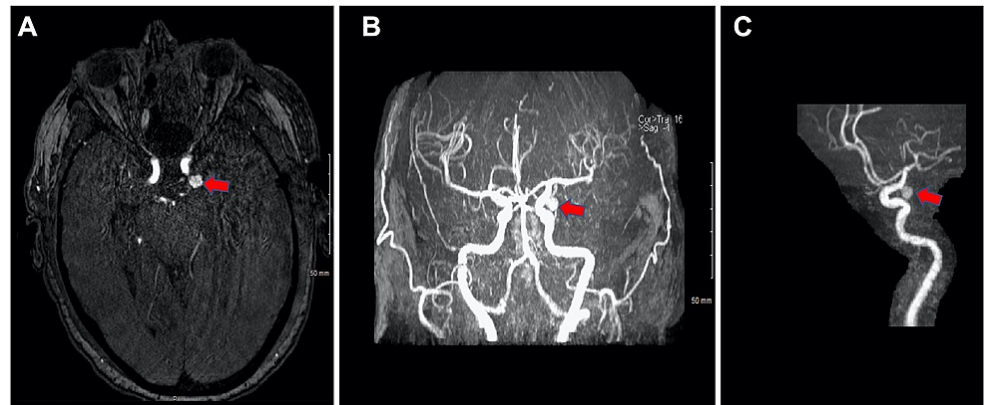
MRA of the Brain Left supraclinoid internal carotid artery aneurysm (red arrows).

Given the patient’s declining neurological exam and radiographic findings, she was taken to the operating room for emergent evacuation of the subdural hematoma. Following surgery, magnetic resonance angiography (MRA) of the brain was performed and revealed a left-sided 6mm posterior communicating artery (PCoA) aneurysm (Figure 2).

**Figure 3:**
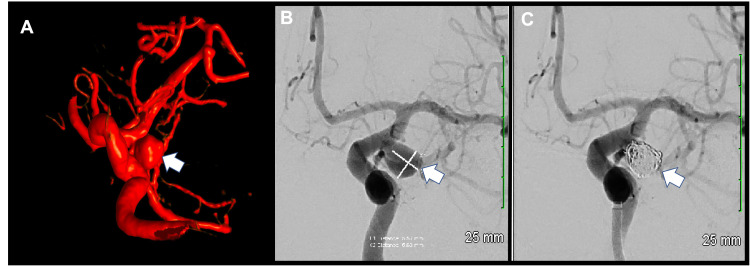
Catheter Angiogram of the Brain A. Three-dimensional rotational subtraction angiogram showing a saccular aneurysm extending laterally from the supraclinoid left internal carotid artery (white arrow). B. Digital subtraction angiogram (DSA) of the left internal carotid artery showing a saccular aneurysm extending laterally from the supraclinoid left internal carotid artery which measures 6 x 6 mm (white arrow), C. Digital subtraction angiogram (DSA) of the left internal carotid artery showing the coiled saccular aneurysm (white arrow).

Urgent coil embolization of the aneurysm was successfully performed via right radial artery access with Raymond-Roy class 2 occlusion (Figure 3).

CT scan of the head ten days after endovascular coiling showed continued improvement in the subdural hematoma and no new intraparenchymal bleeding. In the following days, the patient’s right-sided weakness significantly improved and her sensory examination remained unremarkable with intact deep tendon reflexes. She was subsequently discharged to a rehabilitation facility.

## Discussion

PCoA aneurysms are one of the most common aneurysms encountered by neurosurgeons and neurointerventional radiologists and are the second most common aneurysms overall, making up 25% of all aneurysms and 50% of all ICA aneurysms [1,2]. Not only can these aneurysms present with symptoms of a typical SAH, but they can also present with an isolated oculomotor nerve palsy or a nontraumatic subdural hematoma. The pathogenesis of subdural hematoma development secondary to aneurysm rupture is poorly understood [3]. The most commonly accepted mechanism involves some traumatic insult that causes the rapid accumulation of blood and subsequent breach in the arachnoid mater, followed by adhesion of the arachnoid to the aneurysmal dome thereby inducing a direct rupture of the aneurysm into the subdural space [3,4]. Because of the variation in the anatomy of such aneurysms and their parent arteries, they can be among the easiest or the most difficult to treat, whether the treatment is surgical or endovascular [2]. Gong et al. reported forty cases of subdural hematomas secondary to intracranial aneurysm rupture [1]. Based on their findings, females were slightly more likely than males to be affected (65.9%) with a mean age at presentation of 46.6 years. Aneurysmal locations included the PCoA (39.0%), middle cerebral artery (24.4%), anterior communicating artery (14.6%), and others (12.2%). Subdural hematomas were observed in the cerebral convexity (58.5%), both the convexity and the tentorium (17.1%), and elsewhere (22.0%) [1,4]. Of note, falcine subdural hematomas have been observed with anterior communicating artery aneurysm rupture, as described by Hatayama et al. [5]. Current literature suggests that all other factors being equal, endovascular coiling should be favored for PCoA aneurysm rupture, whereas microsurgical clipping is the treatment of choice in the middle cerebral artery aneurysm rupture [6,7]. Open surgical treatment is typically only indicated when a large hematoma is present and requires emergent evacuation. 

In the presented case, the patient presented with significant neurological deficits and was found to have a subdural hematoma on CT scan of the head. No SAH was reported on the initial non-contrast computed tomography (NCCT) of the brain, however, scant SAH in the horizontal Sylvian fissure could be appreciated in hindsight. Due to hemodynamic instability, further vascular studies were not obtained, and the patient was taken to the operating room for emergent hematoma evacuation. MRA was done and showed a PCoA aneurysm, and the patient was taken for coil embolization of the aneurysm. Alternatively, this procedure could have been done using microsurgical clipping through a pterional craniotomy, but due to her recent craniotomy and subsequent brain inflammation and swelling, the risk was too high. Microsurgical clipping requires a detailed understanding of the aneurysm’s location, and proper configuration is essential to the successful treatment of these lesions. The pterional craniotomy is a highly flexible skull base approach that provides excellent exposure of the anterior cranial fossa, the circle of Willis, and the interpeduncular region [8]. However, this approach is more invasive making it a suboptimal treatment option in this patient who had a recent SAH and ipsilateral craniotomy. Additionally, had the patient been neurologically stable, vascular studies prior to the initial surgery would have revealed the aneurysm and allowed for microsurgical clipping at the time of hematoma evacuation. 

## Conclusions

In rare cases, ruptured intracranial aneurysms can be associated with isolated subdural hemorrhage. Common treatment options include endovascular coiling and microsurgical clipping. However, endovascular surgery is often preferred, especially in aneurysms not amenable to surgical clipping, in poor surgical candidates, and in cases where the anatomy favors an endovascular approach. The patient, in this case, presented with stroke-like symptoms and was found to have a subdural hemorrhage. After emergent craniotomy to evacuate the hematoma, successful endovascular coiling was performed, and the patient was stabilized for further management.
